# Probabilistic topic modeling for the analysis and classification of genomic sequences

**DOI:** 10.1186/1471-2105-16-S6-S2

**Published:** 2015-04-17

**Authors:** Massimo La Rosa, Antonino Fiannaca, Riccardo Rizzo, Alfonso Urso

**Affiliations:** 1 ICAR-CNR, National Research Council of Italy, via Pietro Castellino 111, 80131 Napoli, Italy; 2 ICAR-CNR, National Research Council of Italy, viale delle Scienze Ed.11, 90128 Palermo, Italy

**Keywords:** Probabilistic topic model, ultra short sequence classification, LDA

## Abstract

**Background:**

Studies on genomic sequences for classification and taxonomic identification have a leading role in the biomedical field and in the analysis of biodiversity. These studies are focusing on the so-called barcode genes, representing a well defined region of the whole genome. Recently, alignment-free techniques are gaining more importance because they are able to overcome the drawbacks of sequence alignment techniques. In this paper a new alignment-free method for DNA sequences clustering and classification is proposed. The method is based on *k*-mers representation and text mining techniques.

**Methods:**

The presented method is based on Probabilistic Topic Modeling, a statistical technique originally proposed for text documents. Probabilistic topic models are able to find in a document corpus the topics (recurrent themes) characterizing classes of documents. This technique, applied on DNA sequences representing the documents, exploits the frequency of fixed-length *k*-mers and builds a generative model for a training group of sequences. This generative model, obtained through the Latent Dirichlet Allocation (LDA) algorithm, is then used to classify a large set of genomic sequences.

**Results and conclusions:**

We performed classification of over 7000 16S DNA barcode sequences taken from Ribosomal Database Project (RDP) repository, training probabilistic topic models. The proposed method is compared to the RDP tool and Support Vector Machine (SVM) classification algorithm in a extensive set of trials using both complete sequences and short sequence snippets (from 400 bp to 25 bp). Our method reaches very similar results to RDP classifier and SVM for complete sequences. The most interesting results are obtained when short sequence snippets are considered. In these conditions the proposed method outperforms RDP and SVM with ultra short sequences and it exhibits a smooth decrease of performance, at every taxonomic level, when the sequence length is decreased.

## Background

The study of genomic sequences for classification and taxonomic purposes has a leading role both in microbial identification [1], with important consequences in the biomedical field, and in the classification of living species such as animals or plants, for studies about the biodiversity of different ecosystems [2]. These kinds of analysis are carried out focusing only on a well defined region of the genome, usually referred as barcode genes: for example the 16S rRNA gene for bacteria [3], and the cytochrome c oxidase I (COI) for animals [4]. The first computational approaches with these data were based on sequence alignments and sequence similarities, obtained through the evolutionary distances, with already identified genomic sequences [5]. More recently, novel machine learning and data mining methodologies have been developed. For example clustering algorithms, which are unsupervised techniques able to find groups of similar objects, have been applied for the identification of the taxonomic rank of bacteria isolates. The aim of this approach was to find a correlation between clusters and collections of bacteria belonging to the same taxon (taxonomic category). Clustering techniques have been used considering similarity among gene sequences expressed both in terms of classic evolutionary models [6,7], and in terms of compression-based models [8,9], that derive their theoretic assumption from the information theory concepts of Universal Similarity Metric [10]. The compression-based approaches have been also adopted for the study of phylogenetic relationships among animal species, considering the barcode COI gene [11,12].

Recent alignment-free computational approaches consider genomic sequences as a collection of *k *-mers. A *k *-mer is a small fragment of DNA string of size *k*. In bioinformatics domain a *k *-mer representation has been used in many works. For example, a deep analysis of *k *-mer spectra has been carried out in [13]; a vector representation of DNA sequence using *k *-mers has been adopted for classification task using Support Vector Machines (SVM) [14] in [15,16], and using Neural Gas algorithm [17] in [18]; *k *-mer occurrences in genomic sequences have been considered for training a Naive Bayesian classifier [19,20]. Two of the most accurate sequence classifiers that adopt a *k *-mer representation, as shown in [21], are the RDP classifier [20] and the Simrank algorithm [22]. The RDP tool trains a Naive Bayesian classifier [23] using as input data the frequency occurrence of *k *-mers of a 16S gene dataset; the fitted probabilistic model is then able to predict the taxonomic label of an unknown (unlabeled) sequence. Simrank tool is a search algorithm that employs *k *-mers representation in order to speed up the sequence similarity searches between an unknown query sequence and a repository of tagged 16S genomic strings.

In this work we propose a new computational method for sequence classification based on *k *-mers representation and text mining techniques. If we consider DNA sequences as documents and the related *k *-mers as words, it is possible to extract the most recurrent themes, or topics, shared by the corpus of sequences. Since similar text documents about specific issues, like economy or biology, share the same topics, our thesis is to demonstrate that sequences belonging to the same most recurring themes (topics) have strong similarities among them and belong to the same taxonomic rank. For this reason, our approach is based on the probabilistic topic modeling methodology [24], usually adopted for identification and classification of text documents. Probabilistic topic models, in fact, are algorithms that, given a set of text documents called corpus, extract a group of probability distributions over the words in the documents, i.e. the topics. Our aim is then to learn a probabilistic topic model using this representation, in order to extract the most probable topics from the DNA corpus. The extracted topics will be used to classify unknown test sequences. Apart from text documents, topic models have been also adopted for the analysis of image, audio and music data. In image processing, it is assumed that similar collections of images share the same visual patterns (representing the topics). This way topic modeling has been applied for example for image classification [25], for building image hierarchies [26] and for linking captions and images [27]. In order to infer musical key-profiles of classical music, music files have been considered as text documents, musical notes as words and musical key-profiles as topics [28]. Topic modeling has also been used for audio information retrieval, as in [29]: authors adopted Latent Dirichlet Allocation (LDA) as topic model, and they considered one of the parameter of the fitted model (namely the posterior Dirichlet parameter) as a feature vector in order to perform classification by means of the SVM algorithm. In bioinformatics, topic models have been applied to genomic data by [30], in order to find the topics, representing a genetic signature, belonging to a population with a shared ancestral parent. Moreover authors in [31,32] applied the probabilistic Latent Semantic Analysis (pLSA) topic model [33] in order to predict annotations of Gene Ontology (GO) terms using only the previously available GO annotations.

We carried out experiments on a rich bacterial dataset, more than 7000 sequences, also including ultra-short sequences (length *≤ *50 bp), in order to consider the robustness of the proposed approach with respect to sequence length. Classification results were compared with the ones provided by the RDP classifier and the SVM classifier.

The rest of the paper is structured as follows: the Methods section reports the computational tools used in the paper, with a focus on the probabilistic topic model adopted and our document paradigm for DNA sequences; the Results and discussion section presents the datasets used and the classification results; finally the conclusions are drawn.

## Methods

In this Section we present our computational approaches to the analysis and classification of genomic sequences. After a brief description of probabilistic topic models, we formalize our document paradigm for gene sequences, then we explain our experimental pipelines both for the training and the testing phases.

### Probabilistic topic models

Probabilistic Topic Models are machine learning techniques adopted in text mining field, in order to mine semantic information from a set of documents, called corpus [34,35,24]. Given a document corpus, probabilistic topic models are able to find a group of recurring themes, called indeed *topics*, that are typical of certain classes of documents. For example, financial papers or scientific papers will exhibit different topics according to their specific arguments. Topics are actually probability distributions over the words of the documents. Imagine that we have a fixed vocabulary that is used to generate our document corpus. A topic is a distribution over this vocabulary: for example the economy topic has words about money and trade, and the biology topic has words about life and cells. Assume that these topics are defined before any document is generated: in order to write a document about the impact on the market of a new biologic discover we will use words from both biology and economy topics. The goal of topic modeling is to automatically discover the topics from a collection of documents. More formally, if we assume that all the topics are defined before the documents are created, then each document belonging to a collection can be generated in two steps. First of all a topic is randomly selected according to the probability distribution over topics for the kind of documents we want to generate; secondly a word is randomly chosen with respect to the distribution over the vocabulary for that topic. If we assume that a document *d *is a sequence of *Q *words, *d *= (*w*_1_*, w*_2_*, ..., w_Q_*), the generative model for documents can be expressed by means of the following probability distribution:

(1) $$P\left(w_{i}\right)=\overset{T}{\underset{j=1}{\sum }}P\left(w_{i}|z=z_{j}\right)P\left(z=z_{j}\right)$$

where *P *(*w_i_*) is the probability of the word *w_i _*in a given document; *P *(*z *= *z_j _*) is the probability of choosing a word from topic *z_j _*for the current document; *P *(*w_i_|z *= *z_j _*) is the probability of sampling the word *w_i_*, given the topic *zj *; *T *is the number of topics. Given the words, representing the observable variables, into a corpus of documents, a probabilistic topic model is learned by estimating the topic distributions per document and the words distribution per topic, representing the hidden variables. The number *T *of topics is a model parameter and it has to be fixed a priori. There are several algorithms used to infer a probabilistic topic model. One of the earliest topic model is the Probabilistic Latent Semantic Analysis (pLSA) algorithm [33]. In pLSA, each document is represented as a set of the mixing proportions among the topics, but it is not defined a generative probabilistic model [36]. That means that it is not possible to assign a topic distribution to documents not belonging to the training set. Because of that, in our work we selected the Latent Dirichlet Allocation (LDA) [36] as probabilistic topic model. LDA is one of the simplest algorithm to infer the topics distributions from the generative document model, defined in Eq. 1, and, unlikely pLSA, it provides a fitted model that is able to assign a topic distribution to test documents (i.e. not belonging to the corpus used to train the model) by computing its posterior probability, defined as the conditional distributions of topics given the words in the document. The generative model introduced by LDA is defined as follows. *P *(*w|z*) is represented as a set of *T *multinomial distributions *φ *over all the *W *unique words of the joint set of documents: *P *(*w|z *= *z_j _*) = *φ*(*j*). *P *(*z*) is represented as a set of *D*, the number of documents *d *in the corpus, multinomial distributions *θ *over the *T *topics: *P *(*z *= *z_j _*) = *θ*(*d*). Documents are then generated by first selecting a distribution over topics *θ *from a Dirichlet distribution. The words in the document are generated by selecting a topic *zj *from this distribution and then by selecting a word from this topic, using the distribution *P *(*w|z *= *z_j _*) that is determined from another Dirichlet distribution. More formally, LDA's generative model can be summarized in the following steps:

1 The word distribution *φ *for each topic, representing the probability of a word occurring in a given topic, is set as

(2) φ≈Dirichlet(δ)

where *≈ *means "is distributed as".

2 The proportions *θ *of the topic distribution for the document *d *are set as

(3) θ≈Dirichlet(α)

3 For every word *w_i_*

(a) Select a topic *z_j _≈ *Multinomial(*θ*).

(b) Select a word *w_i _*from a multinomial probability distribution given the topic *zj *: *p*(*w_i_|z_j _, φ*).

More complex topic models, like Pachinko Allocation Model (PAM) [37] and Hierarchical Dirichlet Processes (HDP) [38] were not taken into account. PAM is able to find correlations between topics. In our work, however, we are not interested in inter-topic correlation because we suppose that topics, related to taxonomic ranks in our framework, are independent each other. HDP is an extended version of LDA since it estimates the number of topics. In this work, as explained in section Results and discussion, we are also interested in how classification results vary depending on the number of topics. For this reason we prefer the LDA model because it allows us to select a priori the number of topics for our experiments.

### Document paradigm for gene sequences

**Figure 1:**
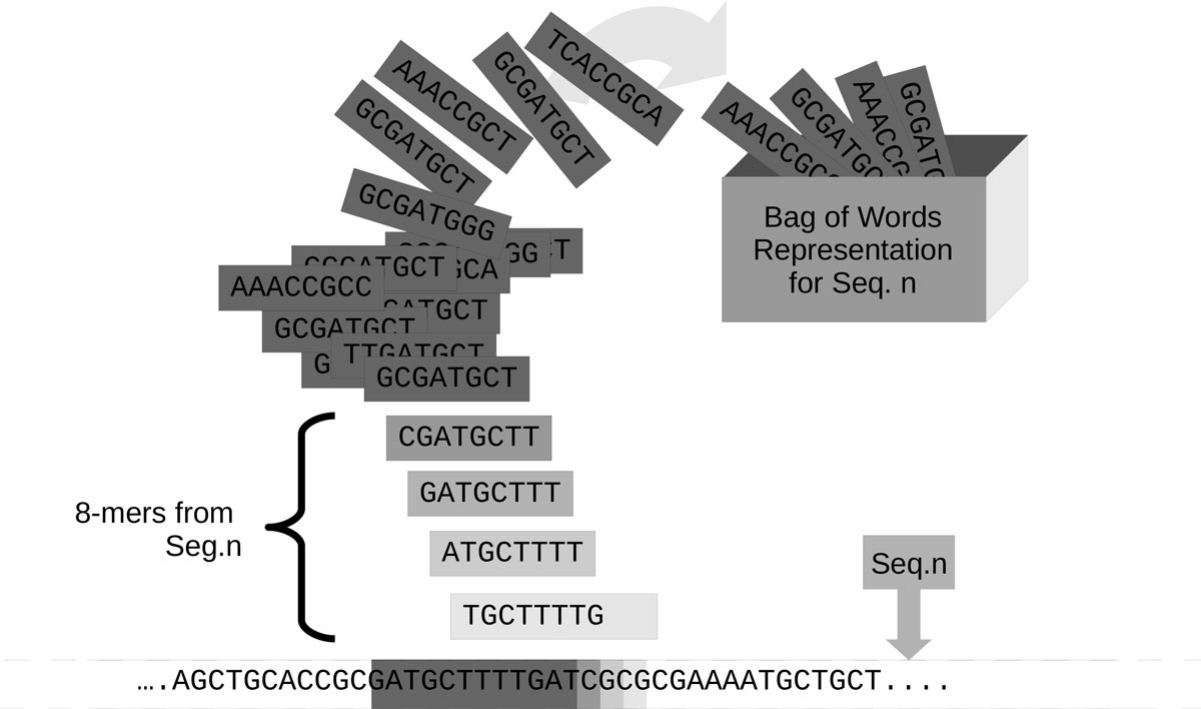
**k-mers decomposition**. By means of a sliding windows of fixed size *k, k *= 8 in this case, it is possible to extract all the overlapping k-mers, representing the words, from a gene sequence.

In this work, probabilistic topic models have been adopted for the study of genomic sequences. Since topic models have been developed for text mining activities, we set up a parallelism between text documents and gene sequences. In our framework, a single DNA sequence, considering only the nucleotide sequence without any header like for instance in the fasta format, represents a document. A dataset of sequences can then be considered as the corpus of the documents. On the other hand, a DNA sequence is composed of only one text string, defined on a fixed alphabet (A, C, G, T). Words can be extracted from gene sequences following the so-called *k *-mer decomposition. As shown in Figure 1, for each sequence in the corpus, all the overlapping *k *-mers can be extracted with a sliding window of fixed length *k *(with *k *= 8 in the figure). The position of a *k *-mer in the original sequence is not taken into account, according to the bag-of-words model used in text analysis. The collection of all the extracted *k *-mers represents, for each sequence, the set of words.

### Finding topics in gene sequences

Using the document paradigm described in "Document paradigm for gene sequences" section, we applied probabilistic topic models to a corpus of nucleotide sequences in order to extract the topics by means of the LDA algorithm. We aim at demonstrating that similar sequences share the same group of most probable topics, so that if it is possible to assign a taxonomic label to those topics, we are able to classify the sequences with respect to their topic distributions. Moreover, using a fitted model, we can also predict the taxonomic rank of an unknown sequence, considering the label of its highest probable topic. The methodologies adopted to assign a label to the topics and to find the most probable topic of a test sequence will be described in the following Sections.

### Training workflow

**Figure 2:**
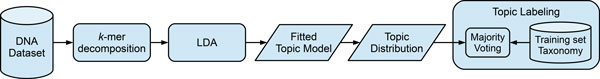
**Training workflow**. From the sequences of the input DNA dataset are extracted the words through the k-mer decomposition; then using the Latent Dirichlet Allocation (LDA) algorithm a probabilistic topic model is learned. The model provides the topic distribution of the input dataset, retrieved from the Ribosomal Database Project (RDP) online repository, and the most probable topics are labeled with a taxonomic rank using a majority voting scheme.

Our proposed procedure for training probabilistic topic models of genomic dataset is shown in the workflow of Figure 2, where round rectangles are processing steps and parallelograms stand for input or output data and models. All the sequences of the DNA corpus are first decomposed with the *k *-mer representation in order to extract their words. Then the LDA algorithm is used in order to infer the probabilistic topic model for a fixed number of topics *T*. In this work we used the LDA implementation provided by the R package *topicmodels *[39]. The LDA model, defined as in section Methods, is fitted using Gibbs sampling [40] and considering parameter values as suggested in [35]. Given a fitted topic model, it is possible to obtain the posterior topic distributions for each sequence of the corpus. For each sequence *di *in the training set, the topic assigned to each training sequence is defined as:

(4) $$z_{j},d_{i}=\text{arg}\underset{j}{\text{max}}P\left(z_{j}|d_{i}\right),with\;j=1,...,T;i=1,...,N$$

where *T *is the number of topics, *N *is the number of sequences in the training set and *P *(*z|d*) is the topic distribution for document.

In order to assign a taxonomic label to each topic, we adopted a majority voting scheme. In fact, we decided to give each topic the taxonomic label belonging to the most of sequences that exhibit that topic with the highest probability. For each topic *z_j _*and considering only the documents *d_i _*assigned to that topic according to Eq. 4, the taxonomic label $l_{z_{j}}$ of topic *z_j _*is defined as:

(5) $$l_{z_{j}}=l_{d_{i}}:i=\text{argma}{\text{x}}_{i}\overset{R}{\underset{k=i+1}{\sum }}f\left(d_{i};d_{k}\right);with\;i=i,\ldots ,R;$$

(6) $$f\left(d_{i},d_{k}\right)=\left\{\begin{array}{cc} 1 & if\;l_{d_{i}}=l_{d_{k}}\\ 0 & if\;l_{d_{i}}\neq l_{d_{k}} \end{array}\right)$$

where $l_{d_{i}}$ is the label of sequence *d_i_*; *R *is the number of sequences belonging to topic *z_j _*; *f *(*d_i_, d_k _*) is a function that is equal to 1 if the label of sequences *d_i _*and *d_k _*are the same, 0 otherwise.

At the end of the training phase, we then obtain a fitted probabilistic topic model and a set of topics representing the taxonomic ranks of the input DNA corpus.

### Testing workflow

**Figure 3:**
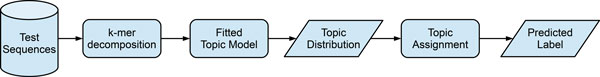
**Testing workflow**. From the test sequences are extracted the words through the k-mer decomposition; then, by means of fitted topic models learned during the training phase, the topic distributions of test sequences are computed. Finally each sequence is assigned to its most probable topic, and, since topics have been labeled during the training phase, the predicted rank for the test sequences is obtained.

The testing procedure of our proposed method works as described in Figure 3. Test sequences are first decomposed into their *k *-mers, then the fitted topic model trained during the learning phase (Figure 2) is used to compute the topic distributions of the test sequences. Afterwards each sequence is assigned to its most probable topic, according to Eq. 5 and considering only the *M *sequences in the test set.

Since, as said in "Training workflow" section, each topic has been labeled with a taxonomic rank during the training procedure, at the end of the testing phase we obtain the predicted taxonomic assignment for the test sequences. The prediction performance of our proposed approach can then be measured using the precision score, defined as:

(7) $$precision=\frac{true\;positives}{true\;positive+false\;positives}$$

where true positives (TP) are correctly classified test sequences, that is their predicted label matches with the topic label; otherwise false positives (FP) represent misclassified test sequences.

## Results and discussion

In this Section we present the 16S bacteria dataset used and we describe both the experiments settings and the results obtained using the probabilistic topic modeling approach for sequence classification. Our results are compared with other two algorithms used for sequence classification: the RDP classifier and the support vector machine classifier.

### Datasets used

We evaluated our approach for gene sequences classification considering bacteria species. For classification and taxonomic studies of bacteria, it is usually considered only a limited part of the genome, about 1200-1400 bp, that is the housekeeping 16S rRNA gene [3]. In our study we arranged a 16S dataset downloading the gene sequences from the Ribosomal Database Project (RDP) repository [41], release 10.32. We chose the four richest phyla, Actinobacteria, Bacteroidetes, Firmicutes, Proteobacteria, and, in order to retain a good quality dataset, we selected the 16S sequences that satisfy the following constraints:

1 type strain, representing reference specimen;

2 size *≥ *1200 bp, considering this way full gene sequences;

3 good quality, according to the quality parameters provided by the RDP repository;

4 NCBI taxonomy, i.e. sequences are labeled with the NCBI taxonomic nomenclature [42].

Moreover we left out unclassified sequences and taxonomic ranks with lesser than ten sequences, in order to obtain a well balanced dataset. Using these criteria, we set up a 16S dataset consisting of 7856 sequences, whose main features are summarized in Table 1.

**Table 1:** Main features of the 16S bacteria Dataset.

**phylum**	**# sequence**	**# class**	**# order**	**# family**
Actinobacteria	2165	1	3	26
Bacteroidetes	760	3	3	11
Firmicutes	1758	4	7	29
Proteobacteria	3173	5	29	66

### Experimental setup

The experiments proposed in this paper, aimed at validating the probabilistic topic modeling approach, represent an expansion and an in-depth analysis of our previous work [43]. There, with a smaller dataset of 3000 sequences, we carried out a series of trials, using a tenfold cross-validation procedure, in order to test how the classification results varied with regards to the number of topics and the dataset composition. We obtained, with *k *-mer size = 8, global results ranging from 99% of precision score at phylum taxonomic level to 80% at family level. In all cases, we noticed that the best scores were reached only when the number of apriori fixed topics is at least equal to the number of different categories of the input dataset. For example, if we want to classify our dataset at order level, we have to train a topic model with a number of topics equal or greater than the number of orders. Of course only in an ideal situation the number of topics matches exactly with the number of categories, in fact in our previous study we obtained better results with a larger number of topics, about two times the number of categories, considering a situation in which each different class covers, in average, two most probable topics. In this work, we enriched that experimental pipeline first of all taking into account a bigger dataset consisting of 7856 gene sequences, described in "Dataset used" section. Moreover, in order to tune the choice of the number of topics, the probabilistic topic models were trained in a hierarchical way. That means we fitted a different topic model at each taxonomic level, for the four different phylum. Considering the Firmicutes phylum, for instance, in order to classify at class level, we trained a model considering an input training set composed of all the Firmicutes sequences. In order to classify at order level, we trained a different topic model for each of the four different classes of Firmicutes phylum (look at Table 1 for info about the number of categories of our bacteria dataset), and so on. As a general rule, we considered, for each topic model a number of topics equal to one time and two times the number of lower categories: if one class has four orders, for that class we trained a topic model with four and eight topics. Once again all the tests have been carried out by means of a ten fold cross-validation procedure.

Unlike our previous work, in this paper we also evaluate the robustness and the generalization ability of our approach with respect to the sequences length. For this reason, we tested our method also with small sized sequences, considering respectively sequence fragments of 400, 200, 100, 50, 40, 25 bp. In this case we submit to the testing workflow a fragment of length *f *(with *f *= 25, 40, 50 and so on) randomly extracted from the full length sequence and we consider the output classification. The need of a robust classifier able to correctly predict the taxonomic rank of small DNA fragments is of fundamental importance in metagenomics applications, where genetic sequences are mainly extracted from environmental species and in many cases ultra short sequences, with size *≤ *50 bp, are available [44].

Classification results, in terms of precision scores (Eq. 7), were compared with other two sequence classifiers: the RDP classifier [20], and the SVM classifier. The former consists of a naive Bayesian classifier trained on a *k *-mer representation of the sequences, the latter works on a vector representation of the gene sequences obtained considering the number of *k *-mers occurrences. We adopted the SVM implementation provided by the R package *e1071 *[45], that allows a simple interface with the well known LIBSVM library [46]. SVM has been run with default parameters and Gaussian Radial Basis kernel.

### Experimental results

**Figure 4:**
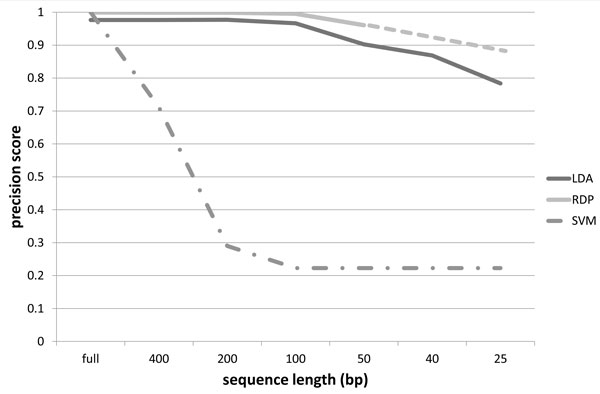
**Precision scores at phylum level**. Precision scores, defined as *true positives*/(*true positives *+ *false positives*), trends as a function of the sequence size (full length, 400 bp, 200 bp, 150 bp, 50 bp, 40 bp, 25 bp), for the Latent Dirichlet Allocation (LDA), Ribosomal Database Project (RDP) and Support Vector Machine (SVM) classifiers at phylum taxonomic rank. The dashed line for the RDP curve represents extrapolated values.

**Figure 5:**
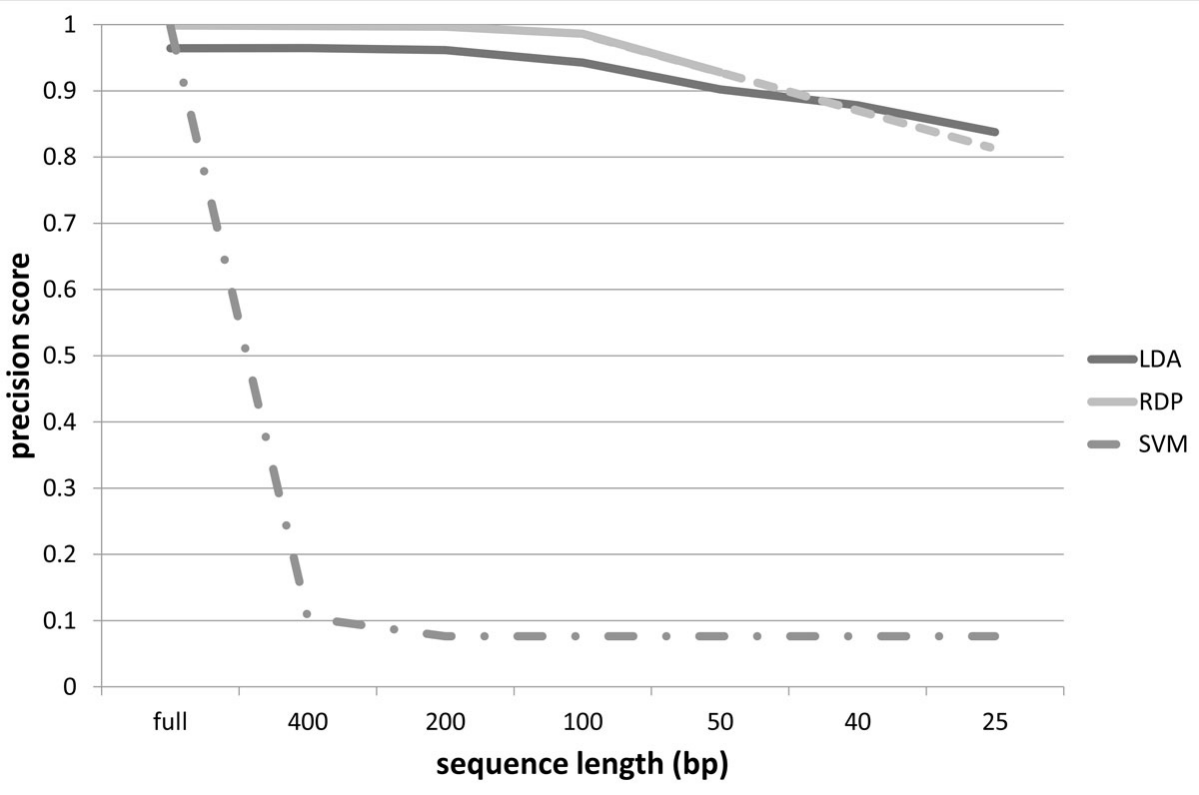
**Precision scores at class level**. Precision scores, defined as *true positives*/(*true positives *+ *false positives*), trends as a function of the sequence size (full length, 400 bp, 200 bp, 150 bp, 50 bp, 40 bp, 25 bp), for the Latent Dirichlet Allocation (LDA), Ribosomal Database Project (RDP) and Support Vector Machine (SVM) classifiers at class taxonomic rank. The dashed line for the RDP curve represents extrapolated values.

**Figure 6:**
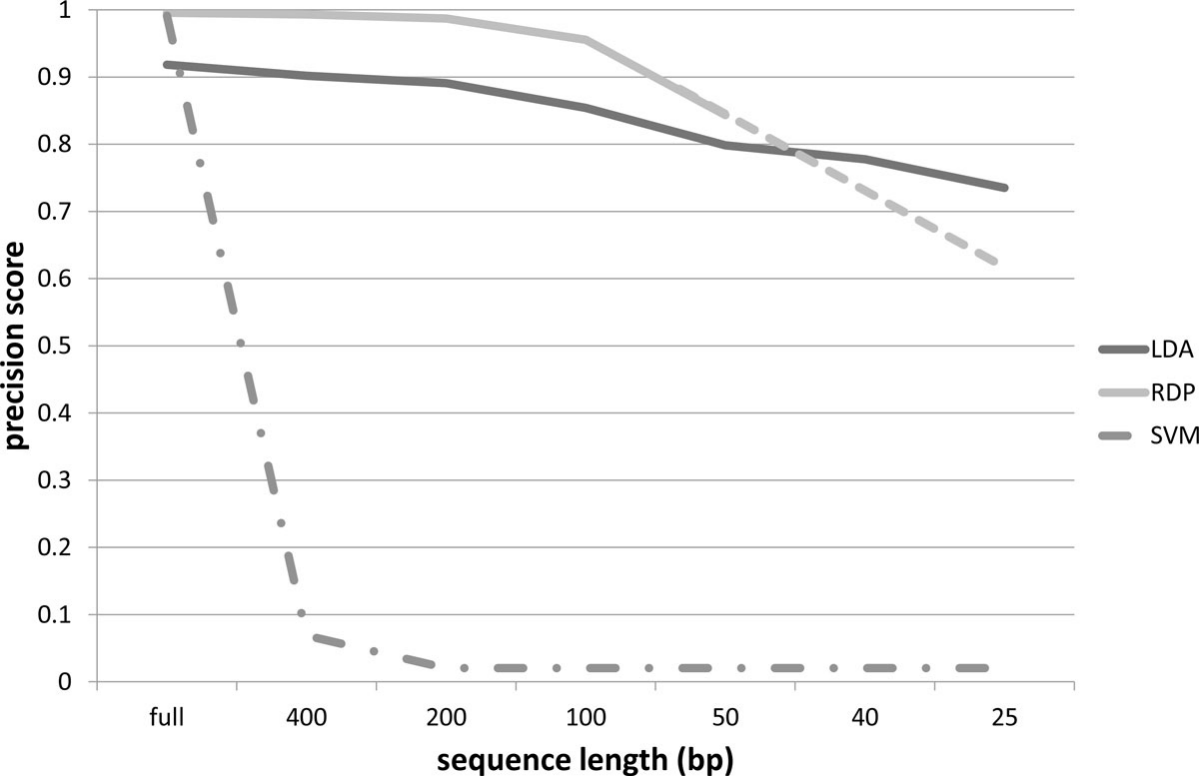
**Precision scores at order level**. Precision scores, defined as *true positives*/(*true positives *+ *false positives*), trends as a function of the sequence size (full length, 400 bp, 200 bp, 150 bp, 50 bp, 40 bp, 25 bp), for the Latent Dirichlet Allocation (LDA), Ribosomal Database Project (RDP) and Support Vector Machine (SVM) classifiers at order taxonomic rank. The dashed line for the RDP curve represents extrapolated values.

**Figure 7:**
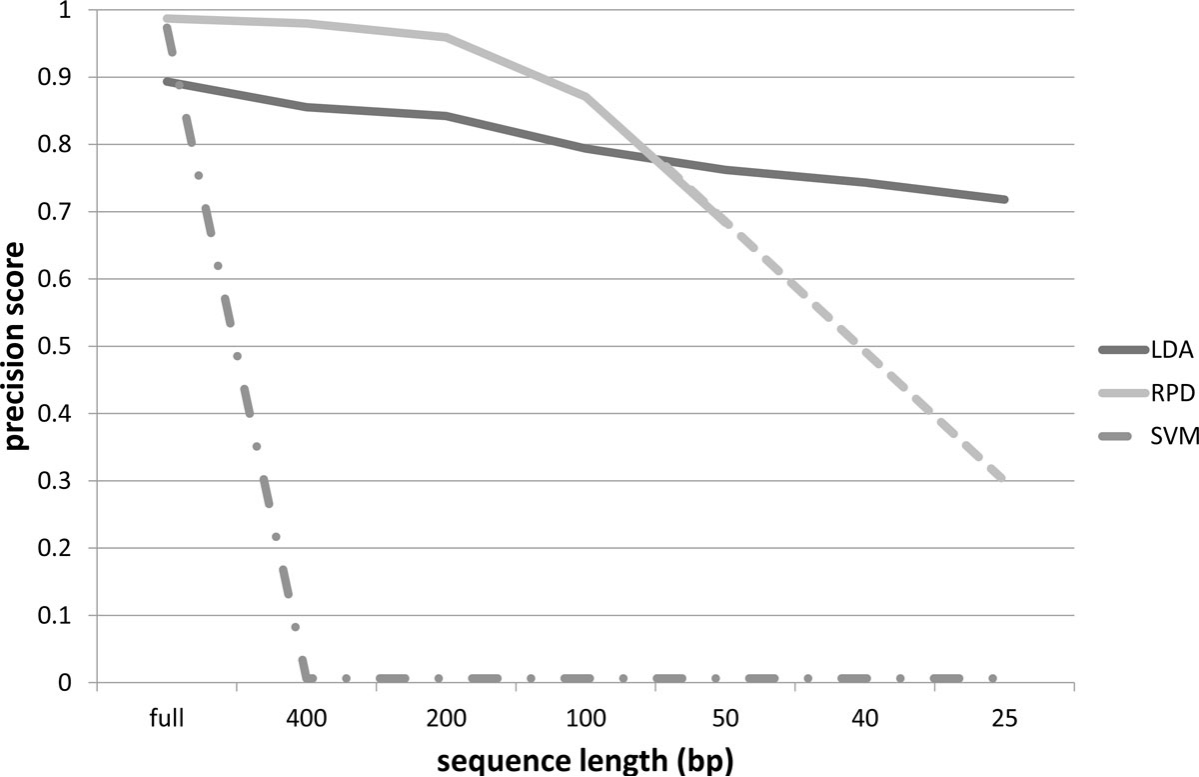
**Precision scores at family level**. Precision scores, defined as *true positives*/(*true positives *+ *false positives*), trends as a function of the sequence size (full length, 400 bp, 200 bp, 150 bp, 50 bp, 40 bp, 25 bp), for the Latent Dirichlet Allocation (LDA), Ribosomal Database Project (RDP) and Support Vector Machine (SVM) classifiers at family taxonomic rank. The dashed line for the RDP curve represents extrapolated values.

The precision scores obtained using our probabilistic topic modeling approach, the RDP classifier and the SVM classifier, for the 16S rRNA dataset described in Section Training dataset, are organized in the charts of Figures 4 to 7. The precision scores are average results obtained by means of a ten fold cross-validation procedure. Each chart shows the score trends as a function of the fragment size (full length, 400 bp, 200 bp, 150 bp, 50 bp, 40 bp, 25 bp) at a different taxonomic rank, from phylum to family. Unfortunately, RDP classifier works only with sequences of at least 50 bp: in fact with fragments of size 40 bp and 25 bp it is unable to provide a classification results. This way precision scores for 40 bp and 25 bp fragments have been linearly extrapolated for the RDP curve. In all the charts, extrapolated values are represented with the dashed line for the RDP curve. From all the charts, it is immediately clear that the SVM classifier provides acceptable precision results, ranging from 99% at phylum level down to 97% at family level, only when applied to full length sequences. In all other situations, the SVM algorithm drops significantly its performances. In fact, with sequence sizes from 400 bp to 25 bp, the SVM looses completely its predictive power, resulting useless when applied to sequence fragments. This behaviour reflects the fact that the vector representation of sequence fragments is quite different from the vector representation of the full sequences composing the training set. SVM, therefore, is not able to generalize the prediction of small sequences. Our approach, briefly called LDA approach from here on, and the RDP classifier show, on the other hand, more robust an significant results. LDA and RDP, in fact, always produce very similar results, at each taxonomic level and for each sequence size, from full length to 50 bp. The LDA's precision scores are slightly lower than the results obtained through the RDP classifier, with an average spread within 10%, and maximum scores greater than 70% in each case. Our LDA approach shows its effectiveness when applied to ultra short fragments, i.e. 50 bp, 40 bp and 25 bp. Considering 50 bp fragments, the LDA and the RDP scores are very close, within 5% of difference, but while the RDP classifier works only with fragment size of at least 50 bp, our LDA approach gives very reliable results, about 70%, even with fragment size of 40 and 25 bp. That means, for example, that with only 25 nucleotides, we are able to predict the family of an unknown sequence with a 70% confidence. Moreover at class, order and family level, our LDA approach not only gives an affordable classification results, but if we compare these scores with the ones extrapolated for the RDP classifier, we obtain higher scores. This behaviour is evident above all in the family case, Figure 7, where the LDA method surpasses the RDP score with 50 bp fragments, with an increase of 11%, and, if we consider the estimated scores of RDP at 25 bp, the performance increment is about 140%. Furthermore in this chart we can observe how the performance decrease of the LDA approach is very smooth, while the RDP classifier shows a rapid decrease, with a performance drop with respect to ultra short sequences (50, 40 and 25 bp).

## Conclusion

In this paper we presented a novel computational approach for gene sequence classification. Using the probabilistic topic models, mainly adopted in text mining applications, we developed a pipeline that, by means of the Latent Dirichlet Allocation algorithm, is able to learn a probabilistic topic model from a dataset of 16S gene sequences. Considering each genomic sequence as a document, our goal is to extract the topics, that are recurring meaningful themes, from the training sequence dataset. On the basis of their topic distributions, our aim is to demonstrate that sequences sharing the same groups of high probable topics belong to the same taxonomic ranks. Classification results, in terms of precision scores, have been compared with the RDP classifier, representing state of the art sequence classifier, and with the SVM general purpose classifier. Experiments were carried out at different taxonomic levels, from phylum to family, and for different sequence sizes, from full length down to 25 bp. The results show our approach reached very similar results, within a 10% spread, compared to RDP and SVM, at every taxonomic level and for full length sequences. The most interesting results were obtained considering the robustness and generalization ability of our method with regards to short sized sequences (from 400 bp to 25 bp). Our approach, therefore, proved very reliable considering full length sequences, with precision scores very close to the ones obtained with RDP and SVM classifiers. Most importantly, it demonstrated its high robustness, with a smooth decrease of performances when applied for classification of ultra short sequences. In the near future, we want to further validate our approach by considering noisy sequences, i.e. "not good" according to RDP repository parameters, and taking into account sequence fragments extracted from different parts of the original sequences. Noisy sequences are interesting because for example in case of environmental species it is possible to obtain degraded sequences. The study of several fragments of the same input sequence can allow us to understand which part of the original sequence carries the most informative content.

## Competing interests

The authors declare that they have no competing interests.

## Authors' contributions

MLR: project conception, implementation, experimental tests, writing, assessment, discussions.

AF: project conception, discussions, assessment, writing.

RR: project conception, discussions, assessment, writing.

AU: project conception, discussions, assessment, writing, funding.

All authors read and approved the final manuscript.

## Declarations

The publication costs for this article were funded by the CNR Interomics Flagship Project "- Development of an integrated platform for the application of "omic" sciences to biomarker definition and theranostic, predictive and diagnostic profiles".

This article has been published as part of *BMC Bioinformatics *Volume 16 Supplement 6, 2015: Selected articles from the 10th International Meeting on Computational Intelligence Methods for Bioinformatics and Biostatistics. The full contents of the supplement are available online at http://www.biomedcentral.com/bmcbioinformatics/supplements/16/S6.
